# Feasibility and radiation induced toxicity regarding the first application of transperineal implementation of biocompatible balloon for high dose radiotherapy in patients with prostate carcinoma

**DOI:** 10.1186/1748-717X-8-82

**Published:** 2013-04-08

**Authors:** Vassilis Kouloulias, Theodoros Kalogeropoulos, Kalliopi Platoni, John Georgakopoulos, George Matsopoulos, Dimitris Chaldeopoulos, Ivelina Beli, Panagiotis Pantelakos, Charalambos Asimakopoulos, John Kouvaris, Nikolaos Kelekis

**Affiliations:** 1 2nd Department of Radiology, Radiotherapy Unit, ATTIKON University hospital, Xaidari, Greece; 2 Institute of Communication and Computer Science, Electrical and Computer Engineering, National Technical University of Athens, Athens, Greece; 3 Urology Department, St. Sawwas Anticancer Hospital, Athens, Greece; 4 1st Department of Radiology, Radiotherapy Unit, Aretaieion University, Athens, Greece; 5 Radiation Oncology, Medical School, National University of Athens, Rimini 1, ATTIKON University Hospital, Xaidari, 12462, Greece

**Keywords:** Acute toxicity, Biocompatible balloon, Prostate cancer, Radiotherapy

## Abstract

**Objective:**

To evaluate the feasibility of the transperineal implementation of biocompatible balloon (Prospace) and the acute toxicity of high dose 3DCRT in patients with localized low risk prostate cancer.

**Materials and methods:**

Between December 2011 and April 2012, fifteen patients were treated with external 3DCRT consisted of 76–78 Gy in 38–39 daily fractions (2.0 Gy/ fraction). Before 3DCRT, we placed the Prospace though the perineum by a minimally invasive procedure in the intermediate space between the rectum and the prostate. The primary study endpoint was the evaluation of acute toxicity according to the EORTC/RTOG radiation toxicity scale. Erectile function was evaluated with the IIEF-5 questionnaire. Rectosigmoidoscopy was performed at baseline, at the end of 3DCRT and 3 months thereafter in order to assess also the rectal toxicity according to Subjective-RectoSigmoid (S-RS) scale. The evaluation of pain related to Prospace implementation was done with the visual analogue score (VAS).

**Results:**

The acute toxicities were as follows: grade I GI toxicity in two patients and for GU toxicity, three patients with grade I of nocturia, four patients with grade I of frequency, two patients with grade I and two patients with grade II of dysouria. The mean score of rectal toxicity according to S-RS score was 1.8(±0.6). The mean VAS score related to Prospace was 1.4(±0.5). Erectile function was unchanged. The Prospace device was found stable in sequential CTs during irradiation.

**Conclusions:**

The implementation of PROSPACE was feasible, while the acute radiation toxicity was low and comparable with IMRT techniques.

## Introduction

Prostate cancer is the most commonly diagnosed neoplasia found in older men, in western world [1]. It represents the second leading cause of oncologic deaths in American men. About one in 20 prostate cancer deaths occur in men ages 55–64, 2 in 10 deaths occur in men ages 65–74 and 7 in 10 prostate cancer deaths occur in men ages 75 and older [2]. One of the standard curative treatments for low risk disease is a radical course of radiation therapy. Radiation therapy is a recognized treatment for PC and high-dose 3DCRTis the recommended standard of care for localized tumours [3].

There is definitely a relationship between dose escalation and response to radiation treatment with radiation induced morbidity to normal surrounding tissues [4,5]. Intensity modulated radiotherapy improves the treatment outcome, sparing the normal surrounding tissues and reducing the acute and late radiation induced toxicity [6-8]. The total dose of radiotherapy that can be delivered through conventional conformal techniques is still limited by the tolerance of surrounding normal tissues, mainly the rectum and the bladder [9].

Levy et al. have already reported on a new balloon made of a biodegradable polymer called Prospace^®^ consisted of poly(lactide-co-epsilon-caprolactone) [10]. Balloon's mechanical and chemical properties were nicely documented both in vitro and in vivo. Prospace was safe and effective for its intended use of separating prostate from the rectum for a desired duration in experimental models [11]. A clinical study was in need to evaluate the safety and efficacy of this device during irradiation in patients with prostate cancer.

Thus, the aim of this study was to evaluate the feasibility of implanted biocompatible balloon as well as the acute GI and GU toxicity for localized low risk prostate cancer patients (cT1-2 N0) undergone high dose radiotherapy. The primary endpoints of this prospective phase II study were the assessment of pain or discomfort related to implementation as well as the monitoring of acute GI and GU radiation induced toxicity. The secondary endpoints were the monitoring of PSA values post irradiation as well as the erectile function after the Prospace implementation.

### Materials and methods

#### Patient characteristics

In a retrospective way, clinical data of 15 patients treated at University Hospital of Athens, Attikon between December 2011 and April 2012, were collected. The pretreatment evaluation included medical history, physical examination, blood profile (including Complete Blood Count, PSA determination, liver function tests, testosterone measurement) and staging exams as CT and MRI of the pelvis. The patient characteristics are summarized in Table 1. All patients had good performance status according to Eastern Cooperative Oncology Group performance score of 0–1. Median follow-up duration was 6 months (range, 3–9 months).

**Table 1:** Patients’ characteristics

	
Median age (range)	71 (65–77)
Mean initial PSA (range)	9.1±0.7 ng/ml (7.2-9.8)
T1	3/15
T2	12/15

#### Inclusion criteria

Eligible patients had histologically confirmed clinical Prostate Cancer Stage T1-2 (according to American Joint Committee on Cancer staging manual, 7th edition, 2010), Gleason score < 7 and prostate-specific antigen (PSA) levels less than 10 ng/mL. All patients had to be medically inoperable for radical prostatectomy due to co-morbidities (cardiac surgery or stent after cardiac mal-function, chronic obstructive lung disease, etc.).

#### Exclusion criteria

Patients were ineligible if they had undergone previous pelvic RT, neoadjuvant androgen ablative therapy or radical prostatectomy, lymph node metastatic involvement, distant metastases or had a hip prosthesis.

#### Balloon implementation

All patients underwent an implementation of Prospace though the perineum. The Prospace Balloon implantation was performed in an outpatient basis under local anesthesia. Prior to the implantation procedure each patient was examined with urine culture to ensure that he has no urine infection, and also was checked for coagulation disorders and stopped any anticoagulant therapy 5 days prior to the implantation date and was replaced with low molecular weight heparin.

The morning of the procedure the patient was administered a per-os antibiotic, preferably such as a fluroquinolone, together with a rectum enema. The Prospace Balloon system packaging consists of a biodegradable inflatable balloon mounted on a deployer, and a balloon delivery echogenic kit consisting of a needle, a dilator and an introducer sheath. The balloon material was a co-polymer of poly lactide acid and epsilon caprolactone which was designed to degrade in situ after 3–6 months from placement. Also a syringe was required to perform skin and fascia anesthesia and to inflate the balloon with warm saline.

**Figure 1:**
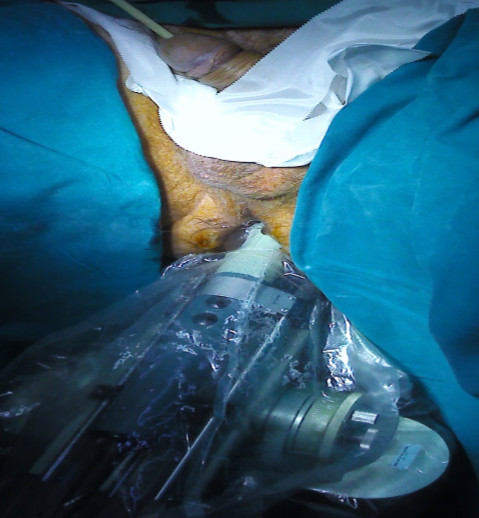
Transrectal ultrasound visualization of the prostate during the balloon implantation procedure.

**Figure 2:**
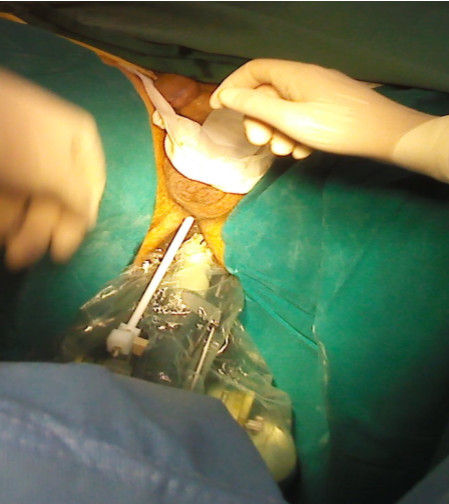
Placement of the introducer sheath under ultrasound guidance.

Placement of the balloon was performed under local anesthesia and continuous TRUS guidance. The patient was placed in lithotomy position and the perineum was scrubbed in a standard manner, while a urethral catheter was inserted and kept in place during the whole procedure. A biplane Transrectal Ultrasound probe (TRUS) was inserted in patient’s rectum and kept in place throughout the whole procedure (Figure 1). Local anesthesia of the perineum was performed by an anesthetic solution injected in the midline approximately 1.5cm above the anal verge. After local anesthesia of the perineum skin and underlying fascia has been achieved, the needle of the delivery kit was inserted in the same location of the local anesthesia and was advanced until the prostate apex under continuous TRUS guidance. From this point, we continue to advance the needle while making hydro dissection with the anesthetic solution, until the needle reaches the prostate base. The hydro dissection with the anesthetic solution produces local anesthesia but also facilitates the separation of the space between the rectum and the prostate and will enable smoother entry and advancement of the introducer sheath later in the procedure. When the needle reaches the correct position, verified by the TRUS, we made a perineal skin and fascia incision, 0,5cm high and 1cm deep around the needle access point. The incision was done to allow room for the dilator and the introducer sheath to be inserted freely into the perineum. Next, while holding the needle in place we advanced the dilator, coupled with the introducer sheath, over the needle towards the prostate base until the designated mark on the needle appears. At this point the dilator tip was aligned with the needle tip and while holding firmly the dilator and introducer sheath in place, we remove the needle. Then, while holding the dilator in place, we continue to advance the sheath over the dilator until the sheath reaches the prostate base as viewed by the TRUS. After verifying the correct position of the sheath in the midline by TRUS, we holded firmly in place the introducer sheath and removed the dilator leaving the introducer sheath in place. Now the introducer sheath acted as a working channel through which the balloon would be introduced. To introduce the balloon, we holded the introducer sheath firmly and we insured that the balloon deployer centering strip was facing upwards while advancing the balloon through the introducer sheath until the mark on the deployer reaches the introducer’s sheath proximal end (Figure 2). Then to withdraw the introducer sheath we holded the balloon deployer firmly and we pulled the introducer sheath all the way back. At this point the balloon was fully exposed in situ and ready for deployment. Once again we verified by TRUS that the balloon was correctly positioned in the midline. We slowly started to fill the balloon with warm saline under continuous TRUS visualization, in order to ensure that during the inflation of the balloon the rectal wall remained at least 3mm thick. The volume of the saline inflated into the balloon, was unique for each balloon, and marked on the package of the balloon, but usually was between 14cc to 17 cc of normal saline. Once the balloon was fully inflated with the designated amount of saline, we detached and sealed the balloon in place by firmly retracting the inflation syringe from the deployer. Now the balloon was firmly positioned in place between rectum and prostate and we could remove safely the deployer and introducer sheath from the patient. We performed a final check of the balloon position by the TRUS as well as by palpation of the rectum to ensure not only that the balloon was in the correct place but also to ensure the integrity of the rectal mucosa. Finally we sutured the perineum incision, if required, and we removed the urethral catheter. The patient was dismissed from the hospital the same day, as soon as he urinates, and was given oral antibiotics for 3–5 days.

The clinical use of PROSPACE was recently approved by the Central Board of Health of the Greek Ministry of Health. Beyond this, all patients signed informed consent about the use of Prospace.

The evaluation of pain or discomfort related to the Prospace implementation was done with the visual analogue score (VAS) [12]. The erectile function was evaluated with the International Index of Erectile Function (IIEF-5) [13].

#### Radiotherapy technique

Each patient underwent a virtual CT-simulation, in supine position, using “knee sponge” to consistently align thighs [14,15]. Patients are instructed to have a full bladder and empty rectum (following a dietary suggestion) during simulation and the whole course of treatment.

For treatment planning, a CT scan covering a region from the first lumbar vertebra to the lower part of the perineum was obtained for each patient. A conventional virtual CT simulation before CT scan was performed to define preliminary isocenter and beam width.

All contouring of target volumes and normal structures (organs at risk-OARs) were performed in the Prosoma Treatment Planning System. Magnetic resonance and computed tomography images were obtained at 3-mm intervals. The CT and MRI were registered by the Prosoma system while corrections were made in the CT-based contouring of the prostate by taking into account the MRI images. CT and MRI images were obtained nearly 4 weeks after balloon implementation in order to avoid the post-implantation oedema. The following structures were delineated: CTV, PTV according to the ICRU criteria [15-17].

The CTV was the prostatic gland; the PTV was obtained by expanding CTV with a margin of 1 cm in each direction, and of 0.7 cm posteriorly [18]. The CTV, PTV and OARs were outlined on all CT slices [19-21]. No patients received pelvic node or seminal vesicles irradiation.

The prescription dose of 76–78 Gy was defined for the 95% isodoses of the PTV. Beams were conformally shaped around the PTV and partial wedging or dynamic Multi Leaf Collimator (MLC) was employed to improve dose homogeneity. To evaluate the dose constraints for normal tissues we used the NCCN 2010 guidelines (http://www.nccn.org), the Radiation Oncology Group (RTOG) GU consensus as reported by Lawton et al. and the QUANTEC report [22,23].

The dose constraints for the OARs are described below:

1. Bladder: V75 <25%, V70 <35%, V65 <50%.

2. Rectum: V75 <15%, V70 <20%, V65 <25%, V60 <35%, V50 <50%.

3. Femoral heads: V50 <5%.

4. Small bowel: V52 = 0%.

5. Penile bulb: Mean dose <50 Gy.

**Figure 3:**
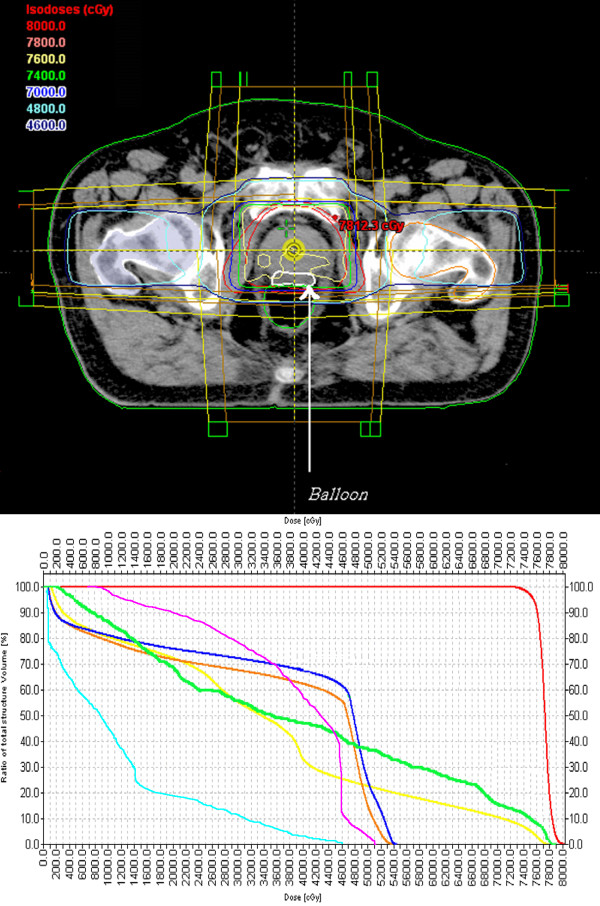
**CT plane with isodose distribution.** The Prospace is outlined in white (arrow). 3**a**: with balloon; 3**b**: without balloon.

The PTV was treated, using a four field technique [24,25]. The total prescribed dose was 76–78 Gy, delivered in 38–39 daily fractions (Monday to Friday) to the whole prostatic gland, given in 2 Gy fractions. Treatments were delivered with 15 MV photon beam generated by a Clinac 2100 C Varian accelerator. A typical dose distribution together with a DVH in a patient after the balloon implemented is shown in Figure 3a, while the dose distribution and the DVH in the same patient before the balloon implementation are shown in Figure 3b.

For the evaluation of stability of Prospace after implementation we used a non-rigid registration technique implemented in four sequential CT scans of the patients during radiotherapy schedule [26].

#### Clinical examination

During the radiation treatment the patients were monitored every week with clinical examination. After the completion of the treatment, the patients were evaluated by a radiation oncologist every month for a three months period of time. GU and GI toxicity were defined according to the RTOG/EORTC acute radiation morbidity scoring system [27]. The rectal toxicity was also assessed with the Subgective-Rectosigmoid Scale using a modified toxicity scale based on LENT-SOMA grading scale [28,29]. The modification took into account the initial LENT-SOMA scale (LENT SOMA tables, 1995), focused on endpoints for acute rectal mucositis together with the scoring system based on the endoscopic terminology of the World Organization for Digestive Endoscopy, as published by the ESGE (European Society for Gastrointestinal Endoscopy) [30,31]. The objective measurements were coming from flexible rectosigmoidoscopy performed at baseline, 1–2 days after the completion of radiotherapy schedule and three months thereafter. The final score was the sum of scores of the 8 items (score=0, if there were no toxicities).

Data at diagnosis (baseline), end of radiation treatment and at all monthly follow up visits, 90 days after finishing radiotherapy have been analyzed in this report. Symptoms occurring in the interval between the start of radiotherapy and 90 days after this time point were classified as “acute”.

#### Statistical analysis

The comparison for PSA values and toxicity levels in terms of paired mean-value statistics, was done with the Wilcoxon non-parametric test. The significance level was set at the level of 0.05. All the statistical test and the descriptive statistics were performed with the SPSS ver 10 software (IL, USA).

## Results

The median follow-up was 6 months. Treatment compliance was excellent. As shown in Figure 3, the D50 for the rectum in terms of a DVH in an irradiated patient was dramatically decreased from 52Gy to 36Gy. According to EORTC/RTOG scale, as shown in Table 2, the acute toxicities were as follows: grade I GI in two patients; for GU, three patients with grade I of nocturia, four patients with grade I of frequency, two patients with grade I and two patients with grade II of dysouria. The mean score of rectal toxicity according to S-RS score was 1.8 (SD±0.6); only grade I toxicity was noted. The results in details concerning the S-RS score are shown in Table 3.

**Table 2:** EORTC/RTOG GI and GU acute radiation induced toxicity

	**Grade 0**	**Grade I**	**Grade II**
*EORTC*-*RTOG scale for lower Gastro*-*intestinal*	None	Increased frequency or change in quality of bowel habits not requiring medication/ rectal discomfort not requiring analgesics	Diarrhea requiring parasympatholytic drugs/mucous discharge not necessitating sanitary pads/ rectal or abdominal pain requiring analgesics
	13/15	2/15	
*EORTC*-*RTOG scale for Genitourinary*			
Nocturia	None	2-3 times	4-6 times
	12/15	3/15	
Frequency	None	Once/2hour	Once/1hour
	11/15	4/15	
Hematuria	None	microscopic	Intermittent (mild/moderate)
	15/15		
Dysouria	None	slight	Moderate
	11/15	2/15	2/15

**Table 3:** Rectal toxicity according to S-RS grading scale at the completion of RT

	**Grade 1**
Subjective	
Tenesmus	Occasional urgency
	1/15
Mucosal loss	Occasional
	2/15
Sphincter control	Occasional
	-
Stool frequency	2-4 per day
	5/15
Pain	Occasional & minimal
	5/15
Objective	
Bleeding	Occult
	-
Mucosa-surface	Localized-spotted congested mucosa
	3/15
Ulceration	Superficial 1 cm^2^
	1/15

Only Grade I acute toxicity was noted.

The implementation of Prospace was feasible. Neither any serious morbidity for the patient nor any difficulty for the urologist who implemented the device was noted. The mean VAS score related to Prospace implementation was minimal at the level of 1.4 (SD±0.5). Some of the patients complained only for a mild discomfort for 24 hours after the implementation.

Acute toxicity was minimal after patients finished RT; at 3 months of follow up, no patient had any GI toxicity score, while two patients remained with grade I GU toxicity (dysouria). The decrease of mean score of EORTC/RTOG acute toxicity at three months compared to the score noted during irradiation was significant (P<0.01, Wilcoxon test). In terms of SRS score, two patients remained with only rectosigmoid findings of grade I toxicity (localized-spotted congested mucosa), showing also a significant reduction compared to the score noted at the completion or RT (P<0.01, Wilcoxon test). As presented in Table 4, the IIEF−5 measurements at baseline and three months thereafter showed no significant differences (P=0.157, Wilxocon test).

**Table 4:** VAS score related to Balloon implementation, S-RS score with subjective and objective findings (rectosigmoidoscopy) at the completion of RT and 3 months thereafter, and PSA values before and after RT

**No**	**VAS score (Prospace)**	**S-RS score (end of RT)**	**S-RS score (3months post RT)**	**IIEF-5 (baseline)**	**IIEF-5 (3months post RT)**	**Baseline PSA**	**Post RT PSA**
1	2	2	0	23	22	8.7	0.3
2	1	2	0	22	23	9.8	0.2
3	2	2	0	22	21	9.1	0.2
4	1	3	1	23	22	9.7	0.1
5	2	1	0	22	22	9.5	0.01
6	1	2	0	22	22	9.6	0.4
7	1	2	0	20	20	7.2	0.1
8	2	2	0	21	22	9.4	0.1
9	1	2	1	22	21	8.7	0.1
10	1	1	0	21	21	8.9	0.01
11	2	1	0	20	20	9.0	1.2
12	1	1	1	20	20	9.6	1.1
13	1	2	1	21	20	8.7	0.9
14	1	2	0	21	20	8.5	0.5
15	2	2	0	23	23	9.8	1.4

The PSA levels in all cases post irradiation were lower than 1.4ng/ml, with a mean value of 0.44 ±0.47. The baseline and post-RT PSA values are presented in Table 4, showing a significant reduction (P<0.01, Wilcoxon test).

Treatment was administered only in cases of GU toxicity: non-steroid anti-inflammatory for dysuria, urgency, frequency, nocturia.

For the registration we used as stable the referential skin markers and the isocenter point. The balloon surface was outlined in CT and its anatomical position was monitored assessing any geographical displacement. The non-rigid registration in sequential CT scans of the pelvis revealed the stability of the Prospace devise, showing displacements only at 3mm maximum.

## Discussion

According to the literature, there is a significant correlation of dose escalation and response to treatment for prostate carcinoma [4,5,8,9]. High dose of conventional fractionated radiation schedules or hypofractionation schemes and associated higher rectal doses have evoked the need for improved protection of the rectum during prostate cancer irradiation [7,9]. Radiotherapy side effects include rectal irritation and bleeding, erectile dysfunction and urinary frequency. Since in conformal radiotherapy, irradiation isodose distribution includes a part of the rectum, displacing irradiated prostate away from anal and rectum, would reduce damage and therefore side effects.

Levy et al. reported on Prospace as an implantable, biodegradable, inflatable, preshaped triangular balloon of commercially used poly(L-lactide-co-epsilon-caprolactone) co-polymer material to provide separation between prostate and rectum [10]. Biocompatibility and degradability of the Prospace in conjunction with local irradiation were evaluated in several in vivo studies [11]. The device was found to be biocompatible in subcutaneously implanted rabbits up to 42 days, in a transperineally implanted dog, up to 12 months and in 8 transperineally implanted pigs, up to 6 months. Since the balloon has been inflated, it remained stable for several months and subsequently the tissues remained separated. In experimental animals, histopathology has shown no systemic or local toxicity. After three months post irradiation evaluation, in pigs that received 15 Gy (3 fractions once per week) the investigators documented the stability of the balloon position without any local or systemic side effects. They also reported that the balloon's preparation ensures no bonding across anatomical interfaces by means of mechanical stability during implantation. In our case the non-rigid registration techniques with sequential CT scans of the pelvis showed also stability of the device [15]. Most studies reported an advantage for IMRT in GI toxicity, attributed to increased conformality of treatment compared with 3DCRT, particularly with regard to volume of rectum treated [3-9]. However, there was some indication that genitourinary toxicity was worse for patients treated with dose escalated IMRT techniques, although most studies did not find a significant treatment effect [3]. In our study the GI toxicity was minimal and equivalent to IMRT techniques, while the GU toxicity was slightly worse in accordance with the reported urinary toxicity from IMRT trials. However we have to emphasize that the technique used was conventional 3D conformal and not IMRT, by means of the sparing of the rectum only due to distance achieved from the irradiated area and definitely of the inability of bladder sparing. Although IMRT is the standard technique for prostate irradiation in many RT departments, the balloon implementation would offer a safe dose escalation, in terms of further decreasing mainly the potential rectal toxicity.

Livi L et al. reviewed 100 patients with localized prostate cancer, receiving 80 Gy with a biphasic technique (3DCRT + IMRT) [32]. The median follow-up time was 12 months, while 18% developed acute Grade 2 rectal toxicity, and no patient experienced acute grade 3 or higher rectal symptoms. Acute Grade 2 urinary symptoms were observed in 44% of the patients. Ruy et al. in another trial with 173 patients reported the toxicity on a 3D-CRT trial for prostate carcinoma with a prescription dose of 79.2Gy [33]. The grade III acute toxicity for bladder or bowel was less than 3%, while 54% of patient presented no or grade I toxicity. Michalski in another study, showed remarkably low grade III toxicity (4%), for patients who received 78Gy at the prostatic gland [34]. Ghadjar et al. reported on 102 patients with 80 Gy prescribed dose, delivered with IMRT technique [35]. The study showed 2% grade II GI toxicity and 43% of less than grade III GU toxicity. Mantzinger et al. in EORTC22991 trial with either 3D-CRT or IMRT for intermediate or high risk prostate cancer, reported 0.8% and 6.3% of grade III GI and GU toxicity, respectively [36]. Al-Mamgani et al. reported a significant lower incidence of acute grade II or higher GI toxicity when IMRT was used instead of 3D-CRT (20% vs 61%, respectively), while no significant difference was noted for GU toxicity between the two techniques [37]. Deville et al. [38] with the use of image-guided intensity modulated radiation therapy for prostate cancer using a daily water-filled endorectal balloon for immobilization, reported maximum GI toxicity of grade 1 and 2 up to 23% and 8%, respectively. In our study the rate of acute toxicity was remarkably low. The reason for this should be the use of Prospace, concerning the GI toxicity and the small field used for the irradiation of the prostatic gland only concerning the GU toxicity (no pelvic fields used). The IIEF−5 questionnaire showed no impact to erectile function related either to Prospace or to radiotherapy, although the position of the balloon was at the anatomical cite of the neurovascular bundle. This fact further indicates the safety of the balloon implementation. However, in our study the number of patients is really low, making the extraction of safe conclusions impossible, even for the evaluation of acute toxicity. Thus we have to emphasize on the implementation method used rather than on the radiation induced morbidity. Moreover, we have to underline the need of a longer follow up, in order to further evaluate the late rectal and urinary radiation induced toxicity.

According to the current literature, PSA nadir predicts biochemical and distant failures after external beam radiotherapy for prostate cancer [39]. Although in our study the follow-up was short, the PSA nadir was obviously achieved as shown in Table 4, meaning that the response to irradiation was excellent. In any case this should be confirmed with more patients and extended follow-up.

At last but not least, we have the impression that the balloon would be appropriate also for interstitial brachytherapy as monotherapy for the prostate cancer [40]. This would have a potential clinical impact in terms of the potential elimination of the already minimum acute rectal toxicity related to brachytherapy. However, concerning the deviation of US, there must be a correction for the filling of the balloon since the content is water and consequently should be different from the surrounding soft tissues. Moreover, there are publications using HDR brachytherapy as boost after external beam RT, reporting a prescription dose of 40-50Gy for the external beam [41-43]. From our point of view, concerning the brachytherapy boost, our technique would allow the escalation of the dose up to 60Gy for the external beam, but this would need a dosimetric study with more patients.

In conclusion, the Prospace implementation is feasible and the radiation induced toxicity especially for the rectum is minimal, equivalent to IMRT techniques. More patients are needed for the confirmation of the results of the present study, which is on-going.

## Abbreviations

RT: Radiotherapy; 3DCRT: 3-Dimentional Conformal Radiotherapy; GI: Gastrointestinal; GU: Genitourinary; ICRU: International Commission on Radiation Units and Measurements; CT: Computed Tomography; MRI: Magnetic Resonance; CTV: Clinical Target Volume; PTV: Planning Target Volume; IIEF-5: 5-item International Index Erectile Function.

## Competing interest

The authors declare that they have no competing interests.

## Authors’ contributions

KV, GJ, CD, BI, PP and AC carried out the target and organ at risk contouring and made the final approving of the planning. KV and GJ made the manuscript draft. KV, CD, BI and AC collect all the required data for the analysis. KV made the statistical analysis. MG made the image registration. KJ and KN made the final editing of the manuscript. All authors read and approved the final manuscript.
